# Assessing the efficacy of intraoperative fluorescence imaging for tumor margin identification in breast conserving surgery

**DOI:** 10.6026/9732063002001927

**Published:** 2024-12-31

**Authors:** Pritika Gnanasekaran, Niraj Balakrishnan, Navaneeth Ranjith, Rakshana Munusamy, J. Naveen Bose

**Affiliations:** 1 Department of Medicine, Global Medical Centre, Salem, Tamil Nadu, India; 2 Department of Surgery, SRM Medical College, Chennai, Tamil Nadu, India; 3 Department of Urology, Caritas Hospital, Kottayam, Kerala, India; 4 Department of Surgery, Madurai Medical College, Madurai, Tamil Nadu, India; 5 Department of Emergency Medicine, Madurai Medical College, Madurai, Tamil Nadu, India

**Keywords:** Breast-conserving surgery, fluorescence imaging, tumor margins, breast cancer, re-excision, intraoperative imaging

## Abstract

Breast-conserving surgery (BCS) aims to remove malignant breast tissue while preserving healthy tissue, with clear margins crucial for reducing recurrence and avoiding additional surgeries. This study evaluated the effectiveness of intraoperative fluorescence imaging (IFI) in achieving negative margins compared to standard intraoperative techniques in 100 retrospectively analyzed patients. IFI-assisted BCS achieved negative margins in 88% of cases versus 65% with standard methods (p < 0.001) and significantly reduced re-excision rates (8% vs. 22%, p = 0.002). Long-term recurrence rates over 12 months were similar between the groups (p = 0.145). These findings suggest that IFI enhances margin visualization, reduces re-excisions and serves as a valuable adjunct to traditional techniques in BCS.

## Background:

Breast-conserving surgery is a cornerstone of breast cancer treatment; it offers the possibility of an effective removal of the tumor while preserving both the aesthetic appearance and functionality of the breast [1]. Positive margins must be minimized since they may lead to recurrence of the tumor locally and subsequent surgical interventions [2, 3]. Positive margins or cancer cells located at the edge of the removed tissue, result in increased recurrence rates and re-excision rates, which results in increased morbidity for patients and healthcare costs [4, 5]. Conventionally, intraoperative margins in BCS have been evaluated by palpation, direct visualization and radiography or ultrasound during surgery. These practices are not extremely sensitive or specific and have resulted in a clinical rate of around 20-30% rescission in BCS [6, 7]. IFI is a novel technology which aims to enhance the visualization of the tumor margins at the time of surgery. It uses fluorescent dyes that attach themselves to the tumor cells, thus providing better capabilities of marking out the tumor from the adjacent normal tissue [8, 9]. Though several studies has demonstrated that the incorporation of IFI results in better accuracy in margin assessment following various surgeries, its utility in BCS is still unexplored. Therefore, it is of interests to assess the role of IFI in the identification of tumor margins, re-excision rates and long-term recurrence in BCS. In this regard, the researchers attempted to establish if IFI can improve surgical outcomes and diminish the need for secondary interventions by comparing IFI with conventional intraoperative techniques [10, 11- 12].

## Methodology:

This retrospective study was conducted between January 2022 and December 2023 on 100 women diagnosed with early-stage breast cancer. All patients underwent breast-conserving surgery: in 50 patients, intraoperative techniques were used in the standard way and in the remaining 50 patients; IFI was used to facilitate the identification of the tumor margin.

## Inclusion criteria:

[1] Female patients aged 30 to 70 years diagnosed with early-stage breast cancer (stages I-II).

[2] Patients undergoing breast-conserving surgery with a single tumor.

## Exclusion criteria:

[1] Patients with multiple tumors or metastatic disease.

[2] Patients unable to tolerate fluorescence imaging agents due to allergies or renal impairment.

## Study design:

## Patients were divided into two groups:

[1] Group A (Standard BCS): 50 patients received traditional intraoperative margin assessment using palpation, visual inspection and intraoperative radiography.

[2] Group B (IFI-Assisted BCS): 50 patients underwent intraoperative fluorescence imaging in addition to standard techniques.

## Intraoperative fluorescence imaging:

Before surgery was initiated, fluorescent dyes were administered intravenously. A near-infrared fluorescence camera system was used by surgeons to obtain images during surgery, which in turn guided the resection in real time and thus helped to identify tumor margins.

## Data collection:

[1] Margin Status: Margins were recorded to be positive if cancer cells were present at or near the edge of tissue otherwise negative if no cancer cells were detected at the margin. This was confirmed by histopathology after surgery.

[2] Re-Excision Rates: Any further surgeries to clear margins were documented.

[3] Recurrence Rates: Follow up for breast cancer recurrence in 12-month follow-up period.

[4] Statistical Analysis: SPSS software, version 26 was used in the analysis of data. In comparing the continuous variables, means ± SD were employed. Categorical variables are presented as percentage of cases in each category Chi-square and t-tests were used to compare the results between groups. The statistical significance level has been considered at a p-value < 0.05.

## Results:

A total of 100 patients were included in this study, of which 50 underwent BCS with traditional technical skills and the other 50 underwent IFI-assisted surgery. Below are the outcomes, which cover margin status, re-excision rates and recurrence. The age, size of the tumor and a grade distribution between the two study groups proved to be not unlike each other, which ensured no baseline characteristics biased the outcome (Table 1). The IFI greatly increased the rate of negative margins, with reduced likelihoods of leaving residual cancer cells (Table 2). The re-excision rate was lower at significantly distinct levels for the group with IFI-assisted procedures; this would indicate that the accuracy of tumor resection is improved by IFI (Table 3). The time to complete the IFI-assisted surgeries was slightly more than for the conventional procedures, indicating that there is additional time expenditure with integrating fluorescence imaging into the surgical procedure (Table 4). The rates of postoperative complications were comparable for both groups, ruling out the possibility that IFI might increase the risk of surgery (Table 5). Recurrence rates at long-term follow-up were comparable in both groups, indicating that the short-term improvements about the margin identification did not influence the long-term outcome (Table 6). Patients of IFI group had a higher level of satisfaction, mainly due to lesser rates of re-excision and better cosmetic outcome (Table 7). The recovery times were similar and thus the use of IFI does not impact recovery time (Table 8). Since it is more expensive for the use of IFI, perhaps the benefit of lower re-excisions will offset some or all of the expense (Table 9). Surgeons felt much more comfortable with their ability to identify margins using IFI and thus it helps to assist in intraoperative decision making (Table 10).

## Discussion:

Although widely accepted for early-stage breast cancers, breast-conserving surgery remains technically challenging with regard to negative margins for both surgeons and patients [13]. Intraoperative fluorescence imaging significantly improved the rate of negative margins during BCS and reduced re-excision rates [14]. The standard method for margin identification, relying purely on palpation and vision, usually misses the microscopic residual disease leading to higher rates of positive margins [15, 16]. IFI, on the other hand, provides real-time visualization of the tumor and makes it easier for the surgeon to achieve clear margins in a further surgery reduction [17]. Outcomes of the current study are comparable with others that had established IFI's effectiveness in various types of surgery like neurosurgery and gastrointestinal surgery [18]. In BCS, more precise resection of tumor tissues allows for a higher probability of minimizing recurrence loco-regional. This is because accuracy has improved re-excision rates in the IFI group [19]. Despite its apparent advantages, IFI has its own limitations.

In this study, operations which involved IFI were more time-consuming to complete and these were costlier than the BCS [20]. The reduction of re-excisions and costs which are associated with redo surgeries can help balance the added upfront expense of the inclusion of IFI in breast surgery for cancer [21]. On the other hand, in terms of postoperative complications, the technology is unlikely to offer any substantial increases and recovery times for patients were equal between both groups [22]. The long-term outcomes, particularly regarding recurrence, were comparable in the IFI and standard BCS groups. This finding thus suggests that whereas IFI does improve surgical short-term results, the long-term cancer control is comparatively not much different compared with standard techniques. Enhanced patient satisfaction and surgeon confidence are proof of the value of the use of IFI as a tool for improving the quality of breast-conserving surgery [23]. The Lumicell (LUM) Imaging System can scan the cavity wall *in vivo* as well as the specimen *ex vivo* which was strength. Also, the auto-fluorescence in the background didn't pose any threat [24].

## Conclusion:

Intraoperative fluorescence imaging greatly enhances the sensitivity of tumor margin detection during breast-conserving surgery, thereby reducing the need for re-excisions and increasing surgeons' confidence. While costs incurred with IFI are obviously heightened and operative times are typically increased, this is somewhat balanced by clinical benefits obtained in terms of achieving negative margins and eliminating repeat surgeries. IFI represents a promising addition to conventional intraoperative methods, offering the surgeon some valuable tools to improve the surgical outcome of breast cancer.

## Figures and Tables

**Table 1:** Baseline characteristics of patients

**Characteristic**	**Group A (Standard BCS)**	**Group B (IFI-Assisted BCS)**	**p-value**
Age (Mean ± SD)	55.2 ± 8.1	54.7 ± 7.8	0.674
Tumor Size (Mean ± SD)	2.1 ± 0.6 cm	2.0 ± 0.5 cm	0.391
Tumor Grade (I:II)	15:25:10	14:26:10	0.812

**Table 2:** Margin status (Positive vs Negative)

**Margin Status**	**Group A (Standard BCS)**	**Group B (IFI-Assisted BCS)**	**p-value**
Negative Margins (%)	65%	88%	<0.001
Positive Margins (%)	35%	12%	

**Table 3:** Re-excision rates

**Group**	**Re-Excision Required (%)**	**p-value**
Group A (Standard BCS)	22%	
Group B (IFI-Assisted)	8%	0.002

**Table 4:** Time to surgery (Minutes)

**Group**	**Mean Time to Complete Surgery (Mean ± SD)**	**p-value**
Group A (Standard BCS)	95 ± 15	
Group B (IFI-Assisted)	110 ± 18	0.005

**Table 5:** Postoperative complications

**Complication Type**	**Group A (%)**	**Group B (%)**	**p-value**
Wound Infection	8%	6%	0.455
Hematoma	4%	2%	0.612
Seroma	6%	4%	0.554

**Table 6:** 12-Month recurrence rates

**Group**	**Recurrence (%)**	**p-value**
Group A (Standard BCS)	4%	
Group B (IFI-Assisted)	2%	0.145

**Table 7:** Patient satisfaction scores (1-5 Scale)

**Group**	**Mean Satisfaction Score (Mean ± SD)**	**p-value**
Group A (Standard BCS)	3.9 ± 0.6	
Group B (IFI-Assisted)	4.5 ± 0.4	0.002

**Table 8:** Time to postoperative recovery (Days)

**Group**	**Mean Recovery Time (Mean ± SD)**	**p-value**
Group A (Standard BCS)	10.5 ± 2.4	
Group B (IFI-Assisted)	9.8 ± 2.1	0.112

**Table 9:** Surgical cost comparison

**Group**	**Average Cost (USD)**	**p-value**
Group A (Standard BCS)	$4,500	
Group B (IFI-Assisted)	$6,200	0.002

**Table 10:** Surgeon confidence in margin identification

**Group**	**Confidence Level (1-5 Scale)**	**p-value**
Group A (Standard BCS)	3.5 ± 0.7	
Group B (IFI-Assisted)	4.6 ± 0.5	<0.001

## References

[1] Mason E.E (2021). Sci Rep..

[2] Byrd B.K (2023). Mol Imaging Biol..

[3] Vorontsov D.A (2022). Sovrem Tekhnologii Med..

[4] Park K.U (2019). Ann Surg Oncol..

[5] Eryilmaz M.A (2011). J BUON..

[6] Kedrzycki M.S (2021). Ann Surg Oncol..

[7] Colakovic N (2018). World J Surg Oncol..

[8] Naffouje S.A (2022). J Med Chem..

[9] Jackson K.M (2023). Ann Surg Oncol..

[10] Wei M (2023). ACS Nano..

[11] Lee E.G (2021). Sci Rep..

[12] Kim J.Y (2016). J Breast Cancer..

[13] Wang J (2023). Thorac Cancer..

[14] Bender H.G, Schnurch HG. (1991). Curr Opin Obstet Gynecol..

[15] Szynglarewicz B (2021). Adv Clin Exp Med..

[16] Smith B.L (2020). Ann Surg Oncol..

[17] Chen Y.J (2016). Sichuan Da Xue Xue Bao Yi Xue Ban..

[18] Barth C.W (2017). Theranostics..

[19] Smith S.L (2014). Int J Radiat Oncol Biol Phys..

[20] Kassem M (2023). Eur J Breast Health..

[21] Mimouni M (2015). Surg Oncol..

[22] Zhang A (2017). Medicine (Baltimore)..

[23] Thill M (2011). Breast..

[24] Li W (2022). Gland Surg..

